# Association between Statin Use and Sensorineural Hearing Loss in Type 2 Diabetic Patients: A Hospital-Based Study

**DOI:** 10.3390/ph14111076

**Published:** 2021-10-25

**Authors:** Hye-Won Han, Jeong Yee, Yoon-Hee Park, Hye-Sun Gwak

**Affiliations:** 1 College of Pharmacy and Graduate School of Pharmaceutical Sciences, Ewha Womans University, Seoul 03760, Korea; smile0808@hanmail.net (H.-W.H.); jjjhello1@naver.com (J.Y.); bagun@amc.seoul.kr (Y.-H.P.); 2 Department of Pharmacy, ASAN Medical Center, Seoul 05505, Korea

**Keywords:** statins, sensorineural hearing loss, tinnitus, type 2 diabetic patients

## Abstract

Statins have emerged as protective agents against sensorineural hearing loss (SNHL) associated with dyslipidemia, but the effects of statins on SNHL are not consistent. The purpose of this study was to investigate the association between statin use and the risk of SNHL using a hospital cohort. This nested case-control study included type 2 diabetic patients over the age of 18 years without a history of hearing loss. Of these, 1379 patients newly diagnosed with SNHL or tinnitus were classified as cases, and 5512 patients matched to the cases based on age, sex, and index year were classified as controls. Chi-squared tests were used to compare categorical variables between the two groups. Odds ratios (ORs) and adjusted odds ratios (AOR) were calculated from univariate and multivariable unconditional logistic regression analyses, respectively. There was a significant difference in the prevalence of statin use between the cases and controls (53.7% vs. 61.2%, respectively; *p* < 0.001). The use of statins in type 2 diabetic patients significantly reduced the risk of SNHL or tinnitus by 24.8% (95% CI 14.2–34.1%, *p* < 0.001) after controlling for confounders. Similar results were found for the association between statin use and SNHL (AOR = 0.706; 95% CI 0.616–0.811, *p* < 0.001). The protective effects of statins against SNHL were consistent regardless of age and sex. The use of statins for type 2 diabetic patients was significantly associated with a reduced risk of SNHL, regardless of age and sex. Further studies are needed, especially large cohort studies, to evaluate the long-term protective effects of statins.

## 1. Introduction

Hearing loss is one of the least visible but most uncomfortable diseases. According to the World Health Organization (WHO), over 5% of the world’s population (432 million adults and 34 million children) has hearing loss and over 900 million people will develop hearing loss by 2050 [1]. Globally, approximately one-third of people over 65 years of age are affected by hearing loss with the prevalence in this age group being greatest in South Asia, Asia-Pacific, and sub-Saharan Africa [1]. Among the Korean population aged 19 years or older, the prevalence of unilateral hearing loss was 9.69% [2]. People with hearing loss experience communication difficulties and suffer from decreased quality of life, isolation, loneliness, and frustration [3]. Furthermore, it is impossible to completely recover to a normal state over a short period, except when hearing aids or cochlear implants are used.

Among the types of hearing loss, sensorineural hearing loss (SNHL) is a condition involving damaged hair cells and the auditory nerve, which is distinguished from conductive hearing loss [4]. Several factors are considered risk factors for SNHL, including aging, noise, chemicals, medications, trauma, and other medical disorders (e.g., autoimmune conditions and infections) [5]. In addition, various metabolic abnormalities, such as type 2 diabetes mellitus (DM) and dyslipidemia, can cause SNHL [6,7].

SNHL is loss of hearing with damage to the inner ear, whereas tinnitus is an auditory sensation in the absence of external stimulus [4,8]. Hearing loss is a known risk factor for tinnitus, and vice versa [9,10,11]. In addition, most hearing-impaired patients experience tinnitus [12,13]. Although the pathophysiological mechanisms of SNHL and tinnitus have not been fully elucidated, shared mechanisms exist between them, including cochlear abnormalities and damaged hair cells [14,15]. In addition, similarly to SNHL, increased lipid levels were significantly related to tinnitus [16].

Statins are widely used as cholesterol-lowering agents through competitive 3-hydroxy-3-methyl glutaryl coenzyme A reductase inhibition. Due to the strong evidence for statins as a treatment option for dyslipidemia, they have emerged as protective agents of hearing associated with dyslipidemia [17,18]. Several studies, however, showed that statins may cause hearing impairment [19,20]. In addition, according to Prayuenyong et al., audiological outcomes were not always correlated with cholesterol levels [17]. As there have been conflicting results regarding statins and hearing loss, additional studies are required. Therefore, the purpose of this study was to investigate the association between statin use and the risk of SNHL or tinnitus, especially for patients with type 2 diabetes mellitus (DM), using a hospital cohort.

## 2. Results

**Figure 1:**
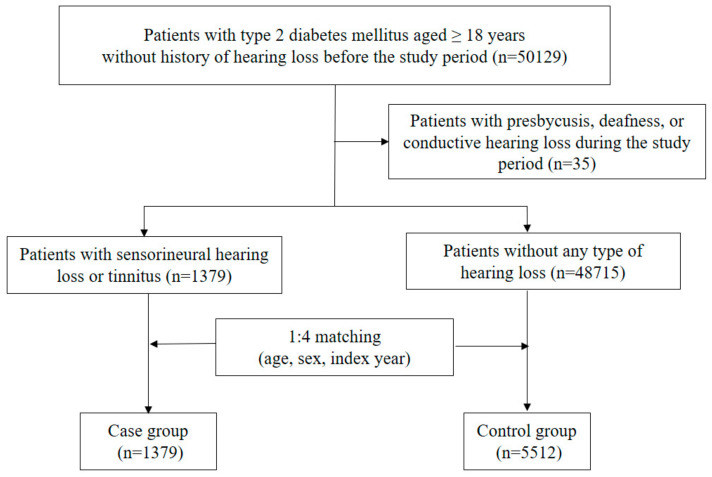
Flowchart for selection of cases and controls.

Among 50,129 patients who were eligible for this study, 35 patients were excluded due to presbycusis, deafness, or conductive hearing loss during the study period (Figure 1). Of 50,094 patients, 1379 cases were newly diagnosed with SNHL or tinnitus during the study period. Approximately three-quarters of cases were first diagnosed by otolaryngologists, and most patients underwent pure tone audiometry and speech audiometry. Cases were matched to 5512 controls based on age, sex, and index year. As shown in Table 1, index year, age, and sex were well balanced between the two groups. Among other factors, a glomerular filtration rate (GFR) less than 30 mL/min/1.73 m^2^, total cholesterol 240 mg/dL or higher, chronic kidney disease, causative diseases of hearing loss, and ototoxic medication use were significant factors for the risk of SNHL or tinnitus.

Table 2 presents the association between SNHL or tinnitus and prior statin use. Statin use was more common in cases than in controls (53.7% vs. 61.2%, *p* < 0.001). When analyzed by statin type, most statins were associated with reduced risk of SNHL or tinnitus, except for fluvastatin and pravastatin. The reductions in the risk of SNHL or tinnitus were 26.2% (95% CI 13.9–36.8%), 36.8% (95% CI 18.0–51.3%), 24.2% (95% CI 9.3–36.6%), and 33.2% (95% CI 16.2–46.7%) for atorvastatin, pitavastatin, rosuvastatin, and simvastatin, respectively.

The results of univariate and multivariable analyses are shown in Table 3. After adjusting for significant factors in the univariate analysis, chronic kidney disease, causative diseases of hearing loss, and the use of ototoxic medications had a 1.7-, 1.4-, or 1.7-fold higher risk of SNHL or tinnitus, respectively. In contrast, statin use showed a protective effect against SNHL or tinnitus; its use was related to a lower risk of SNHL or tinnitus by 24.8% (95% CI 14.2–34.1%). Although total cholesterol 240 mg/dL or higher showed a significantly higher risk of SNHL or tinnitus in the univariate analysis, it failed to remain in the final multivariable analysis model.

Table 4 shows the results of subgroup analysis by sex and age. In the stratified analysis by sex, prior statin use lowered the risk of SNHL or tinnitus in both males (odds ratio [OR]: 0.71; 95% CI: 0.61–0.83) and females (OR: 0.77; 95% CI: 0.65–0.92). For age, the OR for age < 65 years was 0.71 (95% CI: 0.60–0.84) and for age ≥ 65 years 0.76 (95% CI: 0.65–0.90). Neither sex nor age was not found to be an effect modifier (both *p* > 0.05).

Supplementary Tables S1–S3 show the results of secondary analysis where we restricted the case definition to SNHL only. There were 1061 cases and 4241 matched controls. Among these factors, body mass index (BMI) less than 25 kg/m^2^, GFR less than 30 mL/min/1.73 m^2^, chronic kidney disease, causative diseases for hearing loss, and ototoxic medication use were significant factors for SNHL (Supplementary Table S1). Prior statin use was a significant protective factor against SNHL (OR: 0.73; 95% CI: 0.63–0.83); when analyzing by statin type, most statins showed a significantly reduced risk of SNHL, except for fluvastatin and pravastatin (Supplementary Table S2). After adjusting for significant factors from the univariate analysis (Supplementary Table S3), chronic kidney disease, causative disease of hearing loss, and use of ototoxic medications had 1.9-, 1.3-, and 1.6-fold higher risks of SNHL. In contrast, statin use showed protective effects against SNHL; its use resulted in a lower risk of SNHL by 29.4% (95% CI 18.9–38.4%).

## 3. Discussion

This study revealed that the use of statins in patients with type 2 DM significantly reduced the risk of SNHL or tinnitus by 26.3%. Similar results were found in analysis using only SNHL patients as the case group—a 29.4% reduction. Consistent with our results, several studies have reported the protective effects of statins against hearing loss. A survey study reported that statin use was related to a reduced risk of impaired hearing function [21]. Kojima et al. showed that pravastatin significantly improved the mean hearing levels of adults with unilateral sudden SNHL [22]; similarly, atorvastatin was associated with improved tinnitus scores in a cholesterol well-controlled group [23]. However, there have been several conflicting results. Olzowy et al. [24] showed that atorvastatin had no effects on the development of the hearing threshold and did not retard the progression of presbycusis. Another case study reported irreversible hearing loss 18 months after beginning atorvastatin therapy in a 32-year-old man [19]. A population-based study in Taiwan also supported the ototoxic effects of statins; previous statin use was significantly associated with sudden SNHL [20]. This inconsistency may be attributed to the clinical heterogeneity of each study in aspects such as study population, matching variables, and outcome severity. The study in Taiwan selected sudden SNHL cases and sex-, age-, hypertension-, and coronary heart disease-matched controls among patients aged ≥ 40 years [20], whereas this study selected SNHL or tinnitus cases and sex-, age-, and index-year-matched controls among type 2 DM patients aged ≥ 18 years.

One possible mechanism of hearing damage via dyslipidemia involves vascular ischemia of the inner ear artery. Dyslipidemia increases plasma viscosity [25,26], which can trigger stenosis of the cochlear artery, leading to cochlear ischemia and subsequent SNHL [27]. Statins have shown protective effects against SNHL by lowering low-density lipoprotein-cholesterol (LDL-C) and triglycerides, which interfere with the microcirculation of the cochlear vascular system and induce atherosclerotic inflammation. Dyslipidemia may also elevate plasma oxidized low-density lipoprotein levels [28], which mediate the oxidative stress impairment of neurons [29]. In addition to cellular oxidative stress, oxidized low-density lipoproteins stimulate inflammatory cytokines and the expression of adhesion molecules on endothelial cells, which play a role in the initiation of local inflammation in the peripheral nervous system [30]. Consequently, cellular oxidative stress may accompany the initiation of local inflammation, leading to peripheral neuropathies. Oxidative stress may also be involved in the development and progression of hearing loss in the elderly [31], although statins were shown to have antioxidant effects in several studies [32,33,34].

DM, which is one component of the metabolic syndrome, is known as a risk factor for hearing impairment. A cross-sectional study showed that DM was associated with a twofold higher risk of hearing impairment [35]. Another population-based study also showed that the prevalence of SNHL was significantly higher in patients with DM than in those without DM [6]. This can be explained by angiopathy and neuropathy caused by DM [36,37]. In terms of angiopathy, several studies demonstrated that the thickened basal membrane of stria vascularis capillaries observed in DM patients can cause hearing loss [38,39]. When it comes to neuropathy, neuronal atrophy of spiral ganglions and demyelination of the 8th cranial nerve may be related to hearing loss [4,38]. To investigate statin effects excluding the possible influences of type 2 DM, our study population was restricted to type 2 DM patients. The results should be interpreted with caution, especially when applied to non-type 2 DM patients.

Consistent with previous studies [40,41], chronic kidney disease was associated with a higher risk of SNHL or tinnitus. As chronic kidney disease progresses, uremic toxins accumulate, which may cause serial damage in the cochlea [41,42,43]. Hearing loss may result from a decrease in adenosine triphosphatase sodium-potassium pump (Na-K-ATPase) activity [44] and amplitudes of cochlear potentials [45], and further reduction in velocity conduction in auditory nerves [46].

The protective effects of statins against SNHL or tinnitus were found regardless of age and sex. According to a previous study of Koreans [18], medications for dyslipidemia such as statins significantly reduced the incidence of hearing loss in elderly patients aged 60 years and older, but not in those aged under 60 years. Unlike in the previous study, the protective effect of statins against hearing loss was maintained for patients aged 65 years and older as well as those under 65 years.

This study had some limitations. First, we could not evaluate factors that might affect SNHL, such as smoking habits [47,48,49], alcohol consumption [50,51], physical activity, occupations related to noise exposure, and the differences in DM treatment such as drug therapy, exercise, and diet interventions. Second, the assessment of administered medications was not comprehensive. This study did not include the use of non-prescription medications [52,53] (e.g., vitamin B12, folate, coenzyme Q10, etc.) or determine the true compliance of prescription drugs. Third, we did not evaluate the severity of SNHL or tinnitus. Despite these shortcomings, this study provides compelling evidence for the association between statin use and SNHL. Our study is the first study based on a relatively large hospital database, which contains laboratory information for more than 50,000 Korean patients. In addition, this study population consisted of type 2 DM patients, thereby controlling for the confounding effect of DM.

## 4. Materials and Methods

### 4.1. Study Patients

The present study was a single-center, nested case-control study that assessed the association between SNHL and prior statin use among patients who visited the Asan Medical Center, a 2700-bed tertiary care hospital in Seoul, South Korea. This study conformed to the provisions of the Declaration of Helsinki 2013 and was approved by the Institutional Review Board of the Asan Medical Center (IRB No. 2020–0094).

To construct the study cohort, we identified all outpatients aged at least 18 years with a diagnosis of type 2 DM (ICD-10: E11) between 1 January 2015 and 31 December 2019. Individuals diagnosed with all types of hearing loss and tinnitus before the study period (from 1 January 1997 to 31 December 2014) were excluded. To distinguish from other types of hearing loss, based on the most likely mechanisms of SNHL or tinnitus related to statins, patients newly diagnosed with presbycusis (ICD-10: H91.1), deafness (ICD-10: H91.3), or conductive hearing loss (ICD-10: H90.0, H90.1, H90.2) during the study period (between 1 January 2015 and 31 December 2019) were also excluded.

### 4.2. Case and Control Selection

Among our study cohort, cases were defined as all patients newly diagnosed with SNHL (ICD-10: H90, H91, except for H91.1, H91.3, H90.0, H90.1, H90.2) or tinnitus (ICD-10: H93.1) during the study period. We assigned the first visit for the diagnosis of SNHL or tinnitus as the index date for the case. Among the remaining study cohort, controls were randomly matched to cases at a ratio of 4:1 by age, sex, and index year. For cases, the index date was the first diagnosis date; for controls, the index date was the first outpatient visiting date in the matched index year.

### 4.3. Exposure Definition

Statin was the main exposure in this study. Statins used in the hospital included atorvastatin, fluvastatin, pitavastatin, pravastatin, rosuvastatin, and simvastatin. Patients were considered exposed to statin if they had ever been prescribed any statins (Anatomical Therapeutic Chemical classification system (ATC) code: C01AA, C10BX, or C10BA) within one year before the index date.

### 4.4. Potential Confounders

The following data were collected from electronic medical records: patient age, sex, BMI, comorbidities, concurrent medications, and laboratory data. Laboratory data included serum cholesterol, LDL-C, high-density lipoprotein-cholesterol (HDL-C), triglycerides, and hemoglobin A1c (HbA1c).

Based on the findings of previous studies on risk factors for hearing loss [54,55], we considered the following causative diseases of hearing loss as possible confounders (corresponding ICD-10 code in parenthesis): human immunodeficiency virus (B20–24, Z21), cytomegalovirus (B25, P351), rubella (B06, P350), syphilis (A50–53), tuberculosis (A15–19, B90), viral encephalitis (A83–86, B004, B011, B020, B050, B262, B941), viral meningitis (A87, B003, B010, B021, B051, B261), bacterial meningitis (A39, G00), head trauma (S00–09), Meniere’s disease (H810), acoustic nerve disorders (H933), vasculitis (e.g., polyarteritis nodosa, Kawasaki disease, Behçet’s syndrome; M300, M303, M352), and autoimmune disease (e.g., rheumatoid arthritis, systemic lupus erythematosus, antiphospholipid syndrome: M05, M32, D6861).

For ototoxic medication [55,56], we included the following drugs (corresponding ATC code in parenthesis): aminoglycosides (J01G), tetracyclines (J01A), macrolides (J01FA), vancomycin (J01XA01), platinum-based anticancer drugs (L01XA), pyrimidine analogues (L01BC), vinca alkaloids (L01CA), taxanes (L01CD), loop diuretics (C03CA), phosphodiesterase-5 inhibitors (G04BE03, G04BE08, G04BE09, G04BE11), and aspirin (N02BA01).

### 4.5. Statistical Analysis

For the primary analysis, we investigated the effects of statins on SNHL or tinnitus. Chi-squared tests were used to compare categorical variables between two groups. ORs and adjusted odds ratios (AOR) were calculated from univariate and multivariable unconditional logistic regression analyses, respectively. To adjust the confounding factors, multivariable analysis with backward elimination was performed including the factors with a *p*-value < 0.05. Subgroup analyses by sex and age were carried out to evaluate the influence of sex and age on the results. For the secondary analysis, we repeated the primary analysis after restricting cases to those with SNHL only. All statistical analyses were performed with SAS 9.4 (SAS Institute, Cary, NC, USA). A *p*-value < 0.05 was considered statistically significant.

## 5. Conclusions

This study detected a potential association between statin use and SNHL after adjusting for relevant confounders. The results showed that the use of statins for type 2 DM patients was significantly associated with a reduced risk of SNHL, regardless of age and sex. Further studies are needed, especially large cohort studies, to evaluate the long-term protective effects of statins.

## Figures and Tables

**Table 1:** Baseline characteristics of study population for sensorineural hearing loss or tinnitus.

	Case (%) (n = 1379)	Control (%) (n = 5512)	*p*
Index year			1.000
2015	285 (20.7)	1136 (20.6)	
2016	280 (20.3)	1120 (20.3)	
2017	292 (21.2)	1168 (21.2)	
2018	283 (20.5)	1132 (20.5)	
2019	239 (17.3)	956 (17.3)	
Age group (years)			1.000
-29	5 (0.4)	18 (0.3)	
30–39	32 (2.3)	128 (2.3)	
40–49	61 (4.4)	244 (4.4)	
50–59	271 (19.7)	1084 (19.7)	
60–69	483 (35.0)	1932 (35.1)	
70–79	403 (29.2)	1612 (29.2)	
80-	124 (9.0)	494 (9.0)	
Sex			0.998
Male	767 (55.6)	3066 (55.6)	
Female	612 (44.4)	2446 (44.4)	
BMI (kg/m^2^)			0.082
<25	702 (51.5)	2662 (48.9)	
≥25	661 (48.5)	2785 (51.1)	
GFR (mL/min/1.73 m^2^)			<0.001
<30	94 (7.8)	225 (4.7)	
≥30	1115 (92.2)	4604 (95.3)	
Laboratory parameters			
Total cholesterol (mg/dL)			0.039
<240	1144 (96.3)	4692 (97.4)	
≥240	44 (3.7)	125 (2.6)	
LDL-C (mg/dL)			0.749
<160	905 (96.2)	4165 (96.4)	
≥160	36 (3.8)	156 (3.6)	
HDL-C (mg/dL)			0.475
<40	272 (28.5)	1188 (27.3)	
≥40	683 (71.5)	3157 (72.7)	
Triglyceride (mg/dL)			0.899
<500	1124 (99.2)	4845 (99.2)	
≥500	9 (0.8)	37 (0.8)	
HbA1c (%)			0.278
<7.0	526 (51.1)	2204 (49.2)	
≥7.0	503 (48.9)	2272 (50.8)	
Comorbidities			
Coronary heart disease	175 (12.7)	658 (11.9)	0.443
Chronic kidney disease	38 (2.8)	61 (1.1)	<0.001
Causative diseases of hearing loss	92 (6.7)	263 (4.8)	0.043
Ototoxic medications	240 (17.4)	610 (11.1)	<0.001

BMI: body mass index; GFR: glomerular filtration rate; LDL-C: low density lipoprotein-cholesterol; HDL-C: high density lipoprotein-cholesterol; HbA1c: hemoglobin A1c.

**Table 2:** Association between statin use and risk of sensorineural hearing loss or tinnitus by statin types.

	Case (%) (n = 1379)	Control (%) (n = 5512)	Odds Ratio (95% CI)
Prior statin use			
Never	638 (46.3)	2139 (38.8)	1 (ref)
Ever	741 (53.7)	3373 (61.2)	0.737 (0.654–0.830) **
Statin type			
Atorvastatin	291 (21.1)	1322 (23.4)	0.738 (0.632–0.861) **
Fluvastatin	20 (1.5)	46 (1.0)	1.458 (0.856–2.482)
Pitavastatin	76 (5.5)	403 (7.0)	0.632 (0.487–0.820) **
Pravastatin	14 (1.0)	79 (1.3)	0.594 (0.334–1.056)
Rosuvastatin	195 (14.1)	862 (15.3)	0.758 (0.634–0.907) *
Simvastatin	106 (7.7)	532 (9.3)	0.668 (0.533–0.838) **

* *p* < 0.01, ** *p* < 0.001.

**Table 3:** Univariate and multivariable logistic regression analyses for sensorineural hearing loss or tinnitus.

	Crude Odds Ratio (95% CI)	Adjusted Odds Ratio (95% CI)
Total cholesterol (≥240 mg/dL)	1.444 (1.018–2.049) *	
Chronic kidney disease	1.892 (1.306–2.743) ***	1.678 (1.139–2.473) **
Causative diseases of hearing loss	1.427 (1.117–1.823) **	1.393 (1.082–1.794) *
Ototoxic medications	1.693 (1.439–1.992) ***	1.651 (1.393–1.957) ***
Prior statin use	0.737 (0.654–0.830) ***	0.752 (0.659–0.858) ***

Unconditional logistic regression analysis with backward elimination was performed using variables of total cholesterol, chronic kidney disease, causative diseases of hearing loss, ototoxic medications, and prior statin use. * *p* < 0.05, ** *p* < 0.01, *** *p* < 0.001.

**Table 4:** Subgroup analysis by sex and age for the associations between statin use and risk of sensorineural hearing loss or tinnitus.

	Case (%)	Control (%)	Odds Ratio (95% CI)
Sex			
Male (n = 3833)			
Statin non-users	364 (47.5)	1197 (39.0)	1 (ref)
Prior statin users	403 (52.5)	1869 (61.0)	0.709 (0.605–0.831) **
Female (n = 3058)			
Statin non-users	274 (44.8)	942 (38.5)	1 (ref)
Prior statin users	338 (55.2)	1504 (61.5)	0.773 (0.646–0.924) *
Age			
<65 years (n = 3168)			
Statin non-users	330 (52.1)	1099 (43.4)	1 (ref)
Prior statin users	304 (47.9)	1435 (56.6)	0.706 (0.593–0.840) **
≥65 years (n = 3723)			
Statin non-users	308 (34.9)	1040 (41.3)	1 (ref)
Prior statin users	437 (65.1)	1938 (58.7)	0.761 (0.646–0.897) *

* *p* < 0.01, ** *p* < 0.001.

## Data Availability

The data presented in this study are available on request from the corresponding author. The data are not publicly available due to privacy or ethical restrictions.

## Supplementary Materials

The following are available online at https://www.mdpi.com/article/10.3390/ph14111076/s1, Table S1: Baseline characteristics of study population for sensorineural hearing loss, Table S2: Association between statin use and risk of sensorineural hearing loss by statin types, Table S3: Univariate and multivariable logistic regression analyses for sensorineural hearing loss.
