# Supplementary material for: Association between Statin Use and Sensorineural Hearing Loss in Type 2 Diabetic Patients: A Hospital-Based Study
Source: Pharmaceuticals (Basel). 2021 Oct 25;14(11):1076. doi: 10.3390/ph14111076 (PMC8625623; doi:10.3390/ph14111076)
Supplement: Supplementary file 1 [file pharmaceuticals-14-01076-s001.zip › pharmaceuticals-1429478-supplementary.pdf]

Table S1. Baseline characteristics of study population for sensorineural hearing loss.

	Case (%) (n = 1061)	Control (%) (n = 4241)	p
Index year			1.000
2015	213 (20.1)	849 (20.0)	
2016	209 (19.7)	836 (19.7)	
2017	219 (20.6)	876 (20.7)	
2018	229 (21.6)	916 (21.6)	
2019	191 (18.0)	764 (18.0)	
Age group (years)			1.000
-29	2 (0.2)	7 (0.2)	
30-39	23 (2.2)	92 (2.2)	
40-49	44 (4.1)	176 (4.1)	
50-59	180 (17.0)	720 (17.0)	
60-69	356 (33.6)	1424 (33.6)	
70-79	341 (32.1)	1364 (32.2)	
80-	115 (10.8)	458 (10.8)	
Sex			0.997
Male	604 (56.9)	2414 (56.9)	
Female	457 (43.1)	1827 (43.1)	
BMI (kg/m^2)			0.041
<25	547 (52.2)	2038 (48.7)	
≥25	500 (47.8)	2146 (51.3)	
GFR (mL/min/1.73 m^2)			<0.001
<30	80 (8.5)	183 (4.9)	
≥30	857 (91.5)	3532 (95.1)	
Laboratory parameters			
Total cholesterol (mg/dL)			0.147
<240	887 (96.6)	3609 (97.5)	
≥240	31 (3.4)	93 (2.5)	
LDL-C (mg/dL)			0.456
<160	690 (97.0)	3189 (96.5)	
≥160	21 (3.0	116 (3.5)	
HDL-C (mg/dL)			0.929
<40	204 (28.3)	934 (28.1)	
≥40	518 (71.7)	2391 (71.9)	
Triglyceride (mg/dL)			0.516
<500	853 (99.4)	3740 (99.2)	
≥500	5 (0.6)	30 (0.8)	
HbA1c (%)			0.355
<7.0	402 (51.0)	1686 (49.1)	
≥7.0	387 (49.0)	1746 (50.9)	
Comorbidities			
Coronary heart disease	137 (12.9)	530 (12.5)	0.715
Chronic kidney disease	33 (3.1)	45 (1.1)	<0.001
Causative diseases of hearing loss	72 (6.8)	212 (5.0)	0.021
Ototoxic medications	187 (17.6)	480 (11.3)	<0.001

BMI: body mass index; GFR: glomerular filtration rate; LDL-C: low density lipoprotein-cholesterol; HDL-C: high density lipoprotein-cholesterol; HbA1c: hemoglobin A1c

Table S2. Association between statin use and risk of sensorineural hearing loss by statin types.

	Case (%) (n = 1061)	Control (%) (n = 4241)	Odds ratio (95% CI)
Prior statin use			
Never	489 (46.1)	1623 (38.3)	1 (ref)
Ever	572 (53.9)	2618 (61.7)	0.725 (0.633-0.831)**
Statin type			
Atorvastatin	229 (22.2)	1041 (25.1)	0.730 (0.613-0.870)**
Fluvastatin	15 (1.5)	30 (0.7)	1.660 (0.886-3.110)
Pitavastatin	60 (5.8)	317 (7.6)	0.628 (0.468-0.843)*
Pravastatin	12 (1.2)	64 (1.5)	0.622 (0.333-1.162)
Rosuvastatin	150 (14.5)	659 (15.9)	0.755 (0.616-0.927)*
Simvastatin	76 (7.4)	413 (10.0)	0.611 (0.469-0.796)**

*$p < 0.01$, **$p < 0.001$.

Table S3. Univariate and multivariable logistic regression analyses for sensorineural hearing loss.

	Crude odds ratio (95% CI)	Adjusted odds ratio (95% CI)
BMI (≥25)	0.912 (0.845-0.984)*	
Chronic kidney disease	2.153 (1.437-3.225)***	1.870 (1.239-2.822)**
Causative diseases of hearing loss	1.384 (1.050-1.824)*	1.335 (1.008-1.768)*
Ototoxic medications	1.676 (1.394-2.016)***	1.620 (1.341-1.956)***
Prior statin use	0.725 (0.633-0.831)***	0.706 (0.616-0.811)***

Unconditional logistic regression analysis with backward elimination was performed using variables of BMI, chronic kidney disease, causative diseases of hearing loss, ototoxic medications, and prior statin use.

*$p < 0.05$, **$p < 0.01$, ***$p < 0.001$.
